# Irreversible electroporation plus allogenic Vγ9Vδ2 T cells enhances antitumor effect for locally advanced pancreatic cancer patients

**DOI:** 10.1038/s41392-020-00260-1

**Published:** 2020-10-23

**Authors:** Mao Lin, Xiaoyan Zhang, Shuzhen Liang, Haihua Luo, Mohammed Alnaggar, Aihua Liu, Zhinan Yin, Jibing Chen, Lizhi Niu, Yong Jiang

**Affiliations:** 1 Guangdong Provincial Key Laboratory of Proteomics, State Key Laboratory of Organ Failure Research, School of Basic Medical Sciences, Southern Medical University, Guangzhou, 510515 China; 2 Biological Treatment Center, Fuda Cancer Hospital, Jinan University, Guangzhou, 510665 China; 3 Medical Research Centre, The Third Affiliated Hospital, Sun Yat-sen University, Guangzhou, 510630 China; 4 Department of Oncology, Tongji Chibi Hospital, Tongji Medical College, Huazhong University of Science and Technology, Chibi, 437300 China; 5 Department of Respiratory and Critical Care Medicine, Nanfang Hospital, Southern Medical University, Guangzhou, 510515 China; 6 The Biomedical Translational Research Institute, Faculty of Medical Science, Jinan University, Guangzhou, 510632 China; 7 Department of Oncology, Fuda Cancer Hospital, Jinan University, Guangzhou, 510665 China

**Keywords:** Drug development, Tumour immunology

## Abstract

Immunotherapy has limited efficacy against locally advanced pancreatic cancer (LAPC) due to the presence of an immunosuppressive microenvironment (ISM). Irreversible electroporation (IRE) can not only induce immunogenic cell death, but also alleviate immunosuppression. This study aimed to investigate the antitumor efficacy of IRE plus allogeneic γδ T cells in LAPC patients. A total of 62 patients who met the eligibility criteria were enrolled in this trial, then randomized into two groups (A: *n* = 30 and B: *n* = 32). All patients received IRE therapy and after receiving IRE, the group A patients received at least two cycles of γδ T-cell infusion as one course continuously. Group A patients had better survival than group B patients (median OS: 14.5 months vs. 11 months; median PFS: 11 months vs. 8.5 months). Moreover, the group A patients treated with multiple courses of γδ T-cell infusion had longer OS (17 months) than those who received a single course (13.5 months). IRE combined with allogeneic γδ T-cell infusion is a promising strategy to enhance the antitumor efficacy in LAPC patients, yielding extended survival benefits.

**ClinicalTrials.gov ID:** NCT03180437.

## Introduction

Pancreatic adenocarcinoma (PDAC) is a disease associated with poor prognosis, which has an increasing impact on cancer-related mortality worldwide.^1^ According to the American Joint Committee on Cancer (AJCC) criteria, ~40% of PDAC patients (stage III) are diagnosed with locally advanced pancreatic cancer (LAPC), which is defined as the involvement of major vascular structures, leading to unresectable but nonmetastatic diseases.^2,3^

For LAPC patients, gemcitabine-based regimens with or without radiation were the standard treatment, the median OS was 9–11 months.^3^ FOLFIRINOX chemotherapy has prolonged survival; however, the high rates of the toxicity and adverse events (AEs) of chemotherapy limited the treatment promotion.^2^ Currently, immune checkpoint inhibitors (ICI) have demonstrated clinical responses in a number of solid malignancies, but results in pancreatic cancer have been disappointing.^4^ It is reported that the immunosuppressive stroma limited the efficacy of ICIs in PDAC.^5^ In addition, PDAC contains a large number of immunosuppressive cells, i.e., including regulatory T cells (Tregs).^6^ The immunosuppressive microenvironment (ISM) can also inhibit the activity of tumor infiltrating lymphocytes.^7,8^ Therefore, the novel and effective therapies for LAPC patients are needed.

Local ablative therapy such as radiation therapy have been played an important role in LAPC treatment.^9^ Radiofrequency ablation has been described in a few case series of unresectable disease, but the technology is limited to thermal injury to adjacent organs and vessels.^9,10^

Irreversible electroporation (IRE) is a new, nonthermal and minimally invasive technique, which has been approved for LAPC in the FDA and CFDA.^11,12^ IRE could cause tumor cell death by short high-voltage electric pulses.^3,13^ In recent years, several studies have confirmed that IRE is a promising technology in the treatment of LAPC without damaging vessels and nerves.^14–16^ It was reported that IRE promoted gemcitabine entering to PDAC tumor.^17^ IRE may also cause systemic tumor control through changing the structure and composition of the tumor microenvironment (TME), or the priming tumor specific immunity.^2,18^ Thus, IRE could be regarded as a potential immunomodulatory treatment and might induce a large increase of immune cells or parametres after ablation. Moreover, Zhao et al. reported that IRE can potentiate the antitumor efficacy of immunotherapy^5^ Scheffer et al. demonstrated that IRE of LAPC transiently alleviates immune suppression and creates a window for antitumor T-cell activation.^4,19^

In recent years, immune cell therapy has been applied more and more to tumor therapy.^20,21^ In TME, T cells are a key component, and treatment with ICIs or adoptive cell infusion has led to breakthroughs in cancer therapy.^22^ Most clinical application of T cells was centered on αβ T cells (CD4^+^, CD8^+^ T cells), γδ T cells are also important players in cancer immunity.^23^ γδ T cells constitute 0.5–16% of total CD3^+^ cells in the peripheral blood. The human γδ T cells can be activated in a major histocompatibility complex (MHC)-independent manner, which makes it broader antitumor cytotoxicity.^20^ The Vγ9Vδ2 T cell is an important component of immune effector cells that contribute to tumor immune-surveillance against many types of tumors,^24–27^ which indicates that the Vγ9Vδ2-T cell can be used as a promising candidate treatment for LAPC. But up to now, there is only one case report on γδ T cells in cholangiocarcinoma patient,^28^ it demonstrated that allogenic γδ T-cell treatments positively enhanced immune functions, depleted tumor activity, improved quality of life (QOL), and prolonged survival. Based on the promising results above, we designed this clinical trial with to clarify the therapeutical efficacy of IRE in combination with allogenic Vγ9Vδ2 T cells for LAPC patients.

## Results

### Enrolled patients

**Fig. 1:**
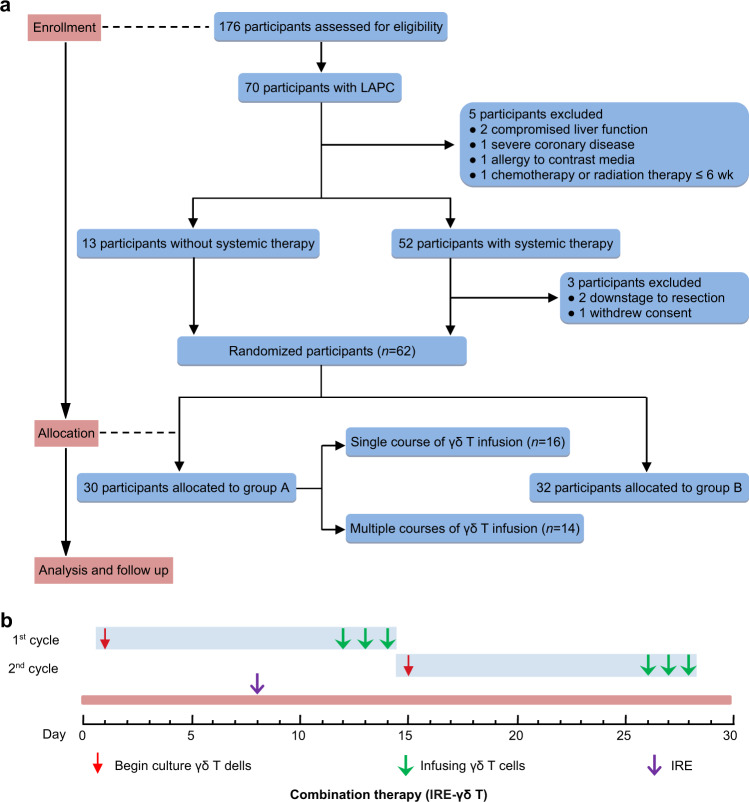
The clinical trial profile and treatment schedule. **a** The clinical trial profile. A total of 176 PDAC patients from 12 countries were screened for enrollment, of which 70 (39.8%) were assessed for LAPC. During screening, five participants were excluded because of compromised liver function (*n* = 2), severe coronary disease (*n* = 1), allergy to contrast media (*n* = 1), and chemotherapy or radiation therapy ≤6 week (*n* = 1). After systemic therapy, three participants were excluded because of downstaging to resection (*n* = 2) and withdrew consent (*n* = 1). Overall, the LAPC patients were screened for the eligibility criteria, and 62 (35.2%) were finally enrolled in the study and assigned randomly to group A (*n* = 30) or group B (*n* = 32). **b** Clinical treatment schedule. Patients in group A received IRE plus one to three courses of allogeneic γδ T cells; one γδ T-cell treatment course was designed to contain two cycles, totaling six γδ T-cell infusions in 28 days, i.e., days 12, 13, and 14 for the first cycle and days 26, 27, and 28 for the second cycle. Patients in group B just received IRE (*n* = 62)

From June 2017 to June 2018, a total of 176 PDAC patients from 12 countries were enrolled after go through a screening process, 70 (39.8%) of them were confirmed for LAPC (Supplementary Table 1). During screening, five patients were eliminated due to compromised liver function (*n* = 2), severe coronary disease (*n* = 1), allergy to contrast media (*n* = 1), and chemotherapy or radiation therapy ≤6 week (*n* = 1). Total three patients were eliminated due to suitable for resection (*n* = 2) and withdrew consent (*n* = 1) after systemic therapy. At last, a total of 62 (35.2%) LAPC patients were enrolled in this trial and randomized into A (*n* = 30) and B (*n* = 32) (Fig. 1a). The clinical characteristics of patients from two groups were generally well balanced (Table 1). Most of the participants received neoadjuvant chemotherapy, including gemcitabine with or without nab-paclitaxel (*n* = 7, 6–16 cycles), FOLFIRINOX (*n* = 37, 2–15 cycles), and others (*n* = 5, 4–6 cycles). The clinical characteristics were also balanced among the patients in group A (Table 1).

**Table 1:** Demographic and disease characteristics of the patients at baseline

Clinical characteristics	All patients		*P* value	Patients in group A		*P* value
	Group A (*n* = 30)	Group B (*n* = 32)		Single γδ T (*n* = 16)	Multiple γδ T (*n* = 14)	
Median age (years)	63 (21–79)	61 (23–80)	0.759	62 (21–80)	64 (23–78)	0.998
Male sex	17 (56.7%)	19 (59.4%)	0.829	9 (56.2%)	8 (57.1%)	0.961
Location			0.885			0.881
Head	19 (63.3%)	21 (65.6%)		10 (62.5%)	9 (64.3%)	
Body	8 (26.7%)	7 (21.9%)		4 (25.0%)	4 (28.6%)	
Uncinate process	3 (10.0%)	4 (12.5%)		2 (12.5%)	1 (7.1%)	
Median tumor diameter (cm)	4	3.9	0.051	4.1	3.9	0.236
Median tumor volume (cm^3^)	38.2	37.5	0.991	37.9	38.3	0.815
Previous surgical therapy			0.863			0.685
None	19 (63.3%)	21 (65.6%)		10 (62.5%)	9 (64.3%)	
Explorative laparotomy	8 (26.7%)	9 (28.1%)		5 (31.3%)	3 (21.4%)	
Others	3 (10.0%)	2 (6.3%)		1 (6.3%)	2 (14.3%)	
Chemotherapy before IRE			0.944			0.993
None	6 (20.0%)	7 (21.9%)		3 (18.8%)	3 (21.4%)	
Gemcitabine with or without nab-paclitaxel	4 (13.3%)	3 (9.4%)		2 (12.5%)	2 (14.3%)	
FOLFIRINOX	18 (60.0%)	19 (59.4%)		10 (62.5%)	8 (57.1%)	
Others	2 (6.7%)	3 (9.4%)		1 (6.3%)	1 (7.1%)	

*IRE* irreversible electroporation, *FOLFIRINOX* 5-fluorouracil, leucovorin, irinotecan, and oxaliplatin

### Safety

**Fig. 2:**
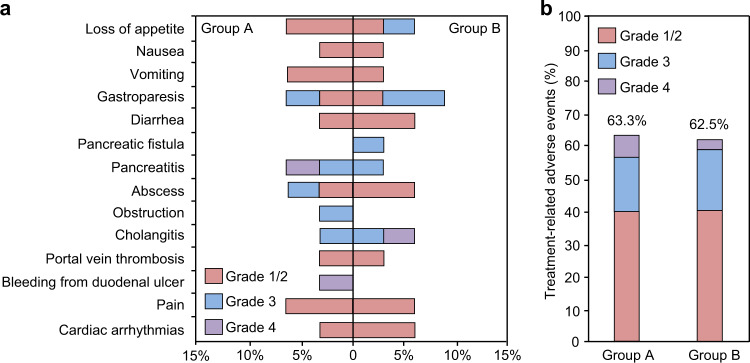
All-cause adverse events in the safety population. **a** All-cause adverse events with a difference of no <5% between the study groups. **b** Proportions of patients with treatment-related adverse events presented by grade. There was no significant difference between the two groups. *n* = 62. *P* > 0.05. Chi-square test

Previous studies verified that immune cell infusion did not cause serious AEs,^28^ so the AEs should be originated from IRE. The main AEs and the proportions of IRE-related AEs during the trial are listed in Fig. 2 and Supplementary Table 3 by grade. The incidence of AEs was similar in two groups (63.3 vs. 62.5%, *P* > 0.05). Overall, there were a total of 14 major and 25 minor AEs happened in 31 of the 62 patients (50%), including 16 participants in group A (53.3%) and 15 in group B (46.9%). Twenty-four patients had one AE, six had two AEs, and one had three AEs. Most minor AEs involved gastrointestinal symptoms, such as nausea, vomiting, loss of appetite, and diarrhea. Other grade I/II AEs were abdominal pain necessitating analgesics, abscesses necessitating antibiotics, and portal vein thrombosis necessitating anticoagulants. Serious AEs (grade III and IV) included biliary obstruction, cholangitis, pancreatitis or pancreatic fistula necessitating antibiotics, severe hematemesis due to bleeding from duodenum necessitating endoscopy, surgical ligation and blood transfusion, and gastroparesis necessitating nasojejunal tube feeding. There was no IRE-related grade V AEs in the trial.

### Carbohydrate antigen 19-9 (CA19-9)

**Fig. 3:**
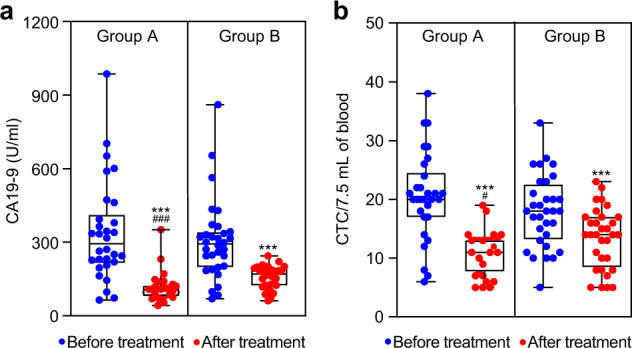
Evaluation of tumor markers before treatment and 90 days after treatment. **a** The level of CA19-9 was quantitated by a chemiluminescent immunoassay. *n* = 62. Data are shown as individual data points with Box and Whiskers graph (bottom: 25%; top: 75%; line: median; whiskers: min to max). Comparison within groups: ***P < 0.001; comparison between groups: ^###^*P* < 0.001. Mann–Whitney test or Wilcoxon test. **b** The number of CD45^−^CK^+^CD326^+^cells (CTCs) was acquired with a FACSCanto^TM^ II. Data are shown as individual data points with Box and Whiskers graph (bottom: 25%; top: 75%; line: median; whiskers: min to max). Comparison within groups: ***P < 0.001; comparison between groups: ^#^*P* < 0.05. Two-tailed Student’s *t* test (*n* = 62)

Before treatment, the mean of CA19-9 was both greater than the normal range. Importantly, despite some efficacy occurred in group B, the level of CA19-9 was markedly reduced in group A (Fig. 3a).

### Circulating tumor cells (CTCs)

By comparison with the IRE group, the number of CTCs was markedly decreased after combination treatment in group A in 7.5 mL of blood (10.77 ± 3.61 vs. 13.41 ± 5.12) (Fig. 3b). A representative change of CTCs from a group A patient is displayed by flow cytometry in Supplementary Fig. 4.

### Immune parameters

**Fig. 4:**
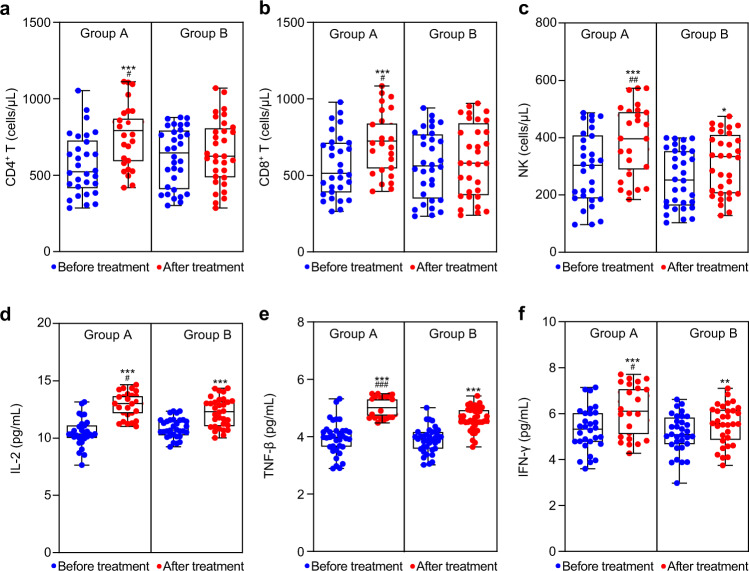
Detection of immune parameters before treatment and 90 days after treatment. **a**–**c** Flow cytometry was performed with the 6-color TBNK reagent to detect lymphocytes in the blood. **d**–**f** Flow cytometry was performed with the Cytometric Bead Array Human Th1/Th2 Cytokine Kit II to detect cytokines in the blood. Data are shown as individual data points with Box and Whiskers graph (bottom: 25%; top: 75%; line: median; whiskers: min to max). Comparison within groups: *P < 0.05; **P < 0.01; ***P < 0.001; comparison between groups: ^#^*P* < 0.05; ^##^*P* < 0.01; ^###^*P* < 0.001. Two-tailed Student’s *t* test (*n* = 62)

We evaluated the immune cells of CD4^+^, CD8^+^, and NK cells using immunofluorescence labeling and flow cytometry. The number of lymphocytes was markedly increased in group A after treatment (Fig. 4a–c). In addition, after combination treatment, the values of Th1 cytokines were markedly increased in group A (Fig. 4d–f).

### Surface receptors

**Fig. 5:**
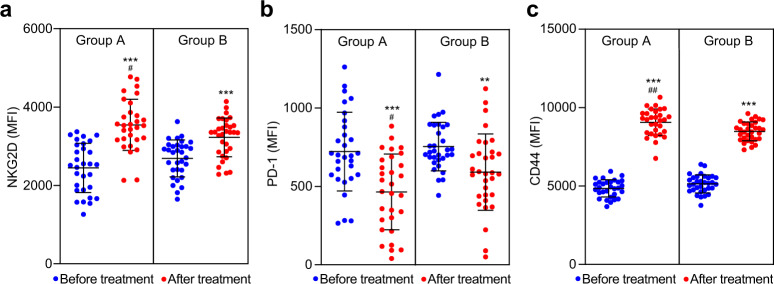
Expression of NKG2D, PD-1, and CD44 before treatment and 90 days after treatment. Surface receptors NKG2D, PD-1, and CD44 on γδ T cells were stained with the monoclonal antibodies of mouse anti-human NKG2D, PD-1, and CD44, then analyzed by flow cytometry with a FACSCanto^TM^ II. MFI mean fluorescence intensity. Data are shown as individual data points with Scatter dot plot (line at: mean with SD). Comparison within groups: **P < 0.01; ***P < 0.001; comparison between groups: ^#^*P* < 0.05; ^##^*P* < 0.01. Two^-^tailed Student’s *t* test (*n* = 62)

As we all know, the receptor, NKG2D, plays an important roles in identifying and killing tumor cells of γδ T cells.^29^ In this study, the level of NKG2D was detected and analyzed. Interestingly, the level of NKG2D receptor was significantly elevated after combination treatment in group A (Fig. 5a). The programmed cell death 1 (PD-1) is an important immunosuppressive molecule, which could initiate the programmed death of T cells via combination of programmed death ligand 1, and then enable tumor cells to obtain immune escape.^30,31^ Surprisingly, the level of PD-1 was significantly downregulated in γδ T cells after combination treatment (Fig. 5b). Meanwhile, the expression of CD44 (surface marker molecule for T-cell activation) was also significantly upregulated (Fig. 5c).

### Performance status (PS) score

**Fig. 6:**
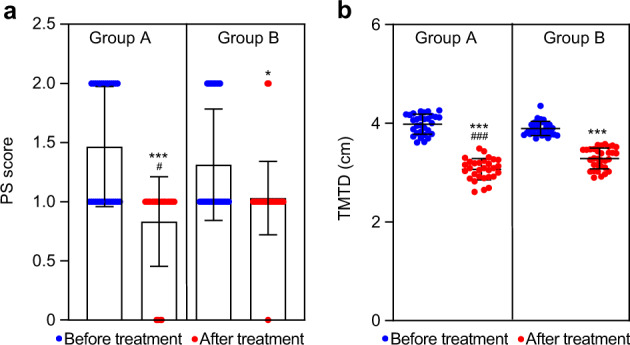
Changes of PS score and TMTD. **a** PS scores were calculated by medical staffs’ observation before and 3 months after treatment. **b** TMTD was evaluated by tumor imaging system before and 3 months after treatment. Data are shown as individual data points with Scatter dot plot (line at: mean with SD). Comparison within groups: *P < 0.05; ***P < 0.001; comparison between groups: ^###^*P* < 0.001. Mann–Whitney test or Wilcoxon test (*n* = 62)

PS score was used to assess the QOL. By comparison with IRE alone, the PS score was significantly decreased after combination treatment (*P* < 0.05) (Fig. 6a).

### Tumor maximum transverse diameter (TMTD)

The TMTDs of patients were listed in Fig. 6b. By comparison with IRE alone, the value of TMTDs was significantly decreased in group A (*P* < 0.05).

### Survival

**Fig. 7:**
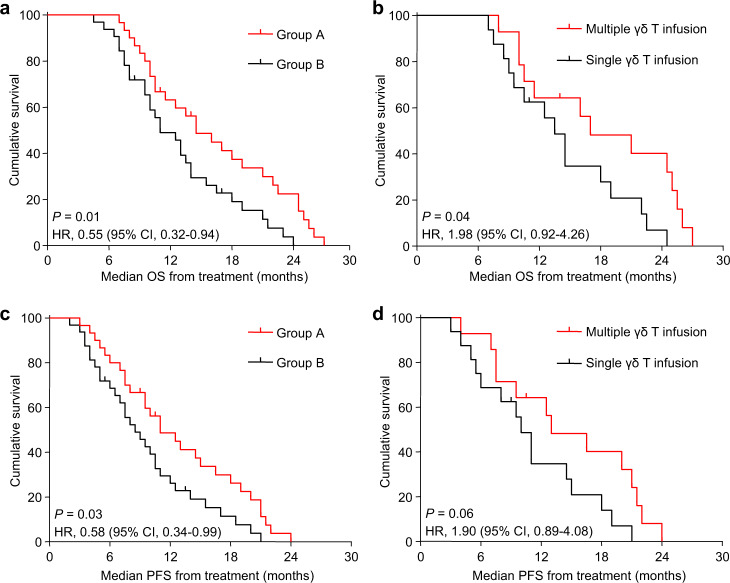
Kaplan–Meier survival curves. **a** Graph showed OS from the time of treatment in total population (*n* = 62). **b** Graph showed OS from the time of treatment in group A patients who received a single course of γδ T-cell infusion or multiple courses of γδ T-cell infusions (*n* = 30). **c** Graph showed PFS from the time of treatment in total population (*n* = 62). **d** Graph showed PFS from the time of treatment in group A patients who received a single course of γδ T-cell infusion or multiple courses of γδ T-cell infusions (*n* = 30)

In group B, the median OS from diagnosis and IRE were 19 months and 11 months; the median OS from diagnosis and combination treatment were 22.5 months and 14.5 months for group A patients, respectively. Obviously, group A patients had a better median OS than that group B (Fig. 7a, Supplementary Fig. 5a). Moreover, by comparison with only receiving a single course of γδ T-cell infusion, the patients who received multiple courses of γδ T cell had a longer median OS (17 months vs. 13.5 months; *P* < 0.05) (Fig. 7b).

For patients in group B, median PFS from diagnosis was 15.5 months, and median PFS from IRE was 8.5 months; the median PFS from diagnosis and combination treatment were 18.5 months and 11 months in group A patients, respectively. Therefore, the median PFS of group A patients was better than group B (Fig. 7c, Supplementary Fig. 5b). However, there was no difference of median PFS between patients who received multiple courses of γδ T-cell infusion and those who received a single course (Fig. 7d). A representative CT image for one patient was shown in Supplementary Fig. 6.

By univariable and multivariable cox regression analyses, we conducted that tumor size was related to worse survival (HR: 13.5, 95% CI: 2.6, 70.6; *P* = 0.002). Patients who received chemotherapy before IRE had a better survival (HR: 5.1, 95% CI: 1.4, 17.9; *P* = 0.012). In addition, patients who with early local progression had markedly worse survival (HR: 0.2, 95% CI: 0.1, 0.5; *P* = 0.001) (Table 2).

**Table 2:** Univariable and multivariable cox regression analysis of OS after treatment

Variable and category	Univariable analysis		Multivariable analysis	
	HR	*P* value	HR	*P* value
Age (years)				
<60	26.5 (24.9, 28.1)	<0.05	N/A	0.594
≥60	20.3 (18.2, 22.3)			
Sex				
Female	26.0 (24.8, 27.2)	<0.05	N/A	0.266
Male	15.0 (13.6, 16.4)			
Tumor size				
<4 cm	29.0 (26.3, 31.7)	<0.05	13.5 (2.6, 70.6)	0.002
≥4 cm	17.0 (13.9, 20.1)			
Chemotherapy before IRE				
Yes	30.0 (26.6, 33.4)	<0.05	5.1 (1.4, 17.9)	0.012
No	17.5 (13.8, 21.2)			
Change in CA19-9 level 3 mo after treatment				
Decrease or no change	26.5 (25.3, 27.7)	<0.05	N/A	0.116
Increase	14.5 (12.6, 16.4)			
Location progression				
≥6 mo or no local progression	24.0 (20.9, 27.0)	<0.05	0.2 (0.1, 0.5)	0.001
<6 mo	11.0 (8.9, 13.1)			

*CA19-9* carbohydrate antigen 19-9, *mo* month, *HR* hazard ratio

## Discussion

For the problem of poor survival in LAPC patients, looking for new strategies to prolong survival is critical. Mounting evidences confirmed that IRE for LAPC was safe and effective.^14,16,32,33^ In our study, 62 participants with LAPC were treated with IRE. The primary result of our study was the median OS in the participants with LAPC from diagnosis was 19 months, which was consistent with the previous result reported by Martin et al.^9^ Ruarus et al. displayed that the OS for LAPC patients was 17 months from diagnosis, due to some patients did not receive any induction chemotherapy before IRE.^3^ Moreover, although IRE therapy was related to certain complications, most of them could be managed via conservative treatment. In recent years, there were emerged some studies about IRE combined chemotherapy for PDAC, which displayed an appropriate extension of survival. However, the toxicity of chemotherapy seriously influenced the QOL of patients. Therefore, novel treatments are needed for LAPC patients.

In recent years, along with the advances in chimeric antigen receptor T-cell and ICIs,^30,34^ immune therapy has become one of the most powerful treatment strategies for malignant tumors. Due to the presence of an immunosuppressive tumor-associated stroma, immunotherapy has only limited efficacy against PDAC.^7,35^ IRE not only killed tumor cells, but also promoted gemcitabine entering to PDAC tumor,^17^ demonstrating a regulation to the stroma. Zhao et al. reported that IRE reversed resistance to immune checkpoint blockade and potentiated the antitumor efficacy of immunotherapy.^5^ Scheffer et al. demonstrated a transient decrease in Tregs and a simultaneous transient increase in activated PD-1^+^ T cells, which was consistent with the transient reduction of tumor-related immune suppression after IRE.^19^ Moreover, a mass of tumor specific antigen released in situ after IRE.^36^ Narayanan et al. suggested that the systemic antitumor immune response triggered by IRE can be enhanced by stimulating the innate immune system with a toll-like receptor-7 (TLR7) agonist and the adaptive immune system with anti-PD-1 checkpoint blockade simultaneously.^4^ Given these, we build a new treatment regimen with local IRE first followed by systemically infusion of γδ T cells.

In TME, T cells are a key component, and treatment with IHI or adoptive cell has led to breakthroughs in cancer therapy.^37,38^ γδ T cells share many qualities with their αβ T-cell counterparts, such as cytotoxic effector functions and pro-inflammatory cytokine production, but one major difference between γδ T cells and αβ T cells is their relative dependence on MHC molecules. Unlike αβ T cells, the low numbers of γδ T cells in mammals slackened the progress on understanding their role in neoplasia.^39^ Fortunately, the major advances of cancer-associated γδ T-cell biology have been displayed in the last few years, such as uncovering their powerful influence on tumors and other immune cells, highlighting their multifaceted role as both anti- and pro-tumor mediators, and so on.^22^ Previous reports have indicated that the Vγ9Vδ2-T cell contributed to tumor immune-surveillance against many types of tumors.^24–27^ Facts proved that allogenic Vγ9Vδ2 T-cell infusion brought more benefits in this study.

In view of the promising results above, we combined IRE with allogenic Vγ9Vδ2 T cells for LAPC patients to observe the efficacy. In comparision with IRE alone, the combination of IRE and allogenic Vγ9Vδ2 T cells significantly enhanced immune function, suppressed tumor growth, and prolonged the survival of LAPC patients in this study. Remarkably, the number of αβ T cells and NK cells was increased after allogenic Vγ9Vδ2 T-cell infusion, and more infusion courses brought more immune cells. This finding was consistent with the previous report by Alnaggar et al.^28^ In addition to increase of lymphocytes, the levels of IL-2, IFN-γ, and TNF-β were also improved, suggesting a stronger antitumor efficacy.^20^ As we all know, NKG2D plays an important role in identifying and killing tumor, IRE plus γδ T cells could markedly upregulated NKG2D. Similarly, PD-1 of γδ T cells was markedly downregulated after IRE plus γδ T-cell therapy. These results are worth noting that combination of IRE and allogenic Vγ9Vδ2 T cells not only potentiate the γδ T-cell lethality but also inhibit the PD-1 expression. Meanwhile, the level of CD44 was significantly upregulated. Collectively, our data suggested that combination of IRE and γδ T cells could strengthen antitumor efficacy. Furthermore, compared with IRE treatment alone, the levels of CA19-9 and CTCs were also markedly declined after combination treatment. Importantly, this is the first report that Vγ9Vδ2 T-cell infusion could reduce the number of CTCs in peripheral blood. Previous reports have confirmed that NK cells caused the reduction of CTCs.^30,40^ Generally speaking, the QOL of PDAC patients is very poor, a good QOL predicts that the development of the tumor will be controlled. Therefore, the other goal of this study was to examine the PS score of LAPC patients. IRE treatment has displayed an improvement in QOL to PDAC patients in the previous reports.^41^ Encouragingly, after receiving the combination of IRE and Vγ9Vδ2 T cells, the patients obtained a better PS score. In this trial, the clinical efficacy of the combination therapy of IRE plus allogeneic γδ T cells was assessed by the changes of above parameters before and after treatment. However, there might be some limitations for the study design without dynamically assessing immunological parameters after treatment.

Above all, IRE plus Vγ9Vδ2 T cells had a better survival than IRE alone in this study. In the present study, the median OS from IRE was 11 months in patients who received IRE only, which was similar to the previous results.^3,16^ Interestingly, the median OS increased to 14.5 months in the patients who received IRE plus Vγ9Vδ2 T cells, which demonstrated that Vγ9Vδ2 T-cell infusion extended the survival time. In addition, by comparison to the patients who never received Vγ9Vδ2 T cell, the patients who received Vγ9Vδ2 T cells had a longer survival, and there was a positive correlation between the OS and the infusion course. The results of regression analysis demonstrated that tumor size, receiving chemotherapy and the time of local progression were the significant predictors of OS.

Our study confirmed that after receiving the Vγ9Vδ2 T cells, the patients emerged an enhanced antitumor effect. Several studies discussed the mechanisms about γδ T cells on antitumor in vivo. Rossi et al. found that boosting γδ T-cell mediated ADCC in follicular lymphoma by PD-1 blockade.^42^ Benyamine et al. demonstrated that BTN3A was a prognosis marker and a promising target for γδ2 T cells based-immunotherapy in PDAC, providing a potential of BTN3A 20.1 mAb toward enhancing Vγ9Vδ2 T-cell antitumor functions.^43^ Chauvi et al. brought new insights into novel immunotherapeutic options for therapy directed at the recurrence of glioblastoma based on allogeneic Vγ9Vδ2 T-cell infusion at the tumor bed.^44^ Capietto et al. displays a new strategy to improve the efficacy of herceptin in HER-2^+^ breast cancer by combination of trastuzumab and herceptin stimulated γδ T cells.^45^ All above studies suggested that the combination of γδ T and other intervention methods could potentiate the antitumor efficacy.

In summary, this clinical trial suggested that IRE combined with γδ T cells enhanced antitumor effects, emerged an extended survival, and provided a new therapeutic strategy for LAPC patients.

## Materials and methods

### Trial design

Patients were divided into group A (IRE plus allogeneic Vγ9Vδ2 T cell), and group B (IRE alone) by a random number table. The combination treatment plan was displayed in Fig. 1b. More broadly, in addition to IRE, patients in group A also received one to three courses of γδ2 T-cell infusion; one course was a 28-day period, containing two infusion cycle. A total of 100 ml venous blood was collected from healthy donors for γδ T-cell expansion.

### Participants

The enrollment criteria included the following: a predicted life expectancy longer than 12 weeks, older than 18 years with either LAPC according to the AJCC criteria, intolerant to chemotherapy or refused to receive chemotherapy, and with an adequate hepatorenal function. The most important exclusion criteria were resectable after chemoradiotherapy, allergy to contrast material, severe coronary disease, a history of level 3 hypertension, myelosuppression, autoimmune disease, not tolerate anesthesia through the trachea, and compromised liver function (e.g., coagulopathy, ascites, portal hypertension and so on), a PS score of >2, and hepatitis B or HIV infection).

### IRE

The IRE was performed using an IRE ablation system (NanoKnife; AngioDynamics, Queensbury, New York). The detailed method about before, during or after IRE was described in Supplementary Data and our previous study.^46^

### Vγ9Vδ2 T cell

Vγ9Vδ2 T-cell expansion and activation is displayed in the Supplementary Data (Supplementary Tables 2, 3; Supplementary Figs. 1–3).^30^

### Follow-up

Contrast-enhanced CT was performed at 3 months after IRE and then every 3 months. Two radiologists interpreted independently to CT result.

### End point

The primary end points were overall survival (OS) and progression-free survival (PFS). Secondary end points were the assessment of safety, i.e., AEs, immune parameters, CTCs, CA19-9, PS score, surface receptor, and TMTD. The detailed description of secondary end points was listed in the method of Supplementary Data.

### Statistical analysis

Safety and efficacy was assessed in all randomly assigned patients. Survival curves were estimated and drawn by Kaplan–Meier analysis. Treatment differences in PFS and OS were assessed by the stratified log-rank. Univariable analysis used to determine prognostic factors. Normality of quantitative data was first analysed using SPSS v 22.0 (IBM, Armonk, NY, USA). For a normal distribution, two-tailed Student’s *t* test was used. For not present a normal distribution, Mann–Whitney test or Wilcoxon test was used. *P* < 0.05 was considered statistically significant.

## Supplementary information

Supplementary Materials
