# Supplementary material for: Irreversible electroporation plus allogenic Vγ9Vδ2 T cells enhances antitumor effect for locally advanced pancreatic cancer patients
Source: Signal Transduct Target Ther. 2020 Oct 23;5:215. doi: 10.1038/s41392-020-00260-1 (PMC7582168; doi:10.1038/s41392-020-00260-1)
Supplement: Supplementary file 1 — Supplementary Materials [file 41392_2020_260_MOESM1_ESM.doc]

Supplementary Materials for

Irreversible electroporation plus allogenic Vγ9Vδ2 T cells enhances anti-tumor effect for locally advanced pancreatic cancer patients

Mao Lin^1, 2#^, Xiaoyan Zhang^1#^, Shuzhen Liang^3^, Haihua Luo^1^, Mohammed Alnaggar^4^, Aihua Liu^5^, Zhinan Yin^6^, Jibing Chen^2*^, Lizhi Niu^2, 7*^, Yong Jiang^1*^

Correspondence to: [18922210657@163.com](mailto:18922210657@163.com)

**This PDF file includes:**

Materials and Methods

Figures. S1 to S6

Tables S1 to S4

**Materials and Methods**

**Vγ9Vδ2 T cell preparation and infusion**

Vγ9Vδ2 T cells were prepared under good manufacturing practice (GMP) conditions using clinical-grade reagents. Briefly, interleukin-2 (IL-2) and zoledronate (Novartis, Nuremberg, Germany) were used to co-stimulate expansion and activation of Vγ9Vδ2 T cells from peripheral blood mononuclear cells (PBMCs) *in vitro* according to the manufacturer’s protocol. In detail, 100 ml of blood was donated by a donor who had passed health examination including check for infectious diseases. Then, PBMCs were isolated from the blood of healthy volunteers following a standard Ficoll-Paque density gradient centrifugation protocol.[^1^](#_ENREF_1) Approximately 3 × 10^7^ isolated PBMCs were suspended in 40 ml 10% RPMI-1640 medium supplemented with IL-2 (10 ng/ml) and zoledronate (50 μM) and cultured in a T175 culture flask at 37°C in a 5% CO_2_ atmosphere at day 1. From day 5, IL-2 was renewed every 2 days at the same concentration and cells were kept at a density of 1 × 10^6^ cells/ml until day 12. On day 7, the cultured cells were counted, if the cell number was more than 6 × 10^7^, the cells were split into 2 culture flasks. On day 9, the cultured cells in each flask were split into 3 flasks, and cell quality control inspection was conducted to certify the cell quality (Supplementary Table 3). Then, the cultured cells were added with fresh γδ T cell culture medium to a final volume of 200 mL on day 11.

On day 12, cells were counted for total cell number, which could reach 4-5 × 10^8^,[^2^](#_ENREF_2) and checked for release criteria (Supplementary Table 4). γδ T cells before expansion and on day 12 after expansion were stained with PE- or FITC-coupled antibodies against Vδ2 (Miltenyi Biotec, Bergisch Gladbach, Germany, ^#^REA771) or CD3 (^#^REA613), respectively, followed by analysis by flow cytometry with a CD3^+^Vδ2^+^ gating strategy. Approximately 1.5 × 10^8^ γδ T cells were harvested from two T175 culture flasks and transferred into a 200 mL transfusion bag for intravenous infusion at a concentration of 1 × 10^6^ cells/mL, which was adjusted by a mixed solution of 150 mL saline and 5 mL human serum albumin. The rest cells were cultured with fresh medium and harvested for daily infusion on days 13 and 14. After infusion within 4 h, the patients rested for 30 min. The preparation and infusion process of γδ T cells is shown in Supplementary Figure 1.

**Cytotoxicity assay**

To assess the cytotoxicity of Vγ9Vδ2 T cells, pancreatic cancer cell line BxPC-3 (ATCC, Manassas, USA) was used as the target cell. 2 × 10^5^ cells/mL BxPC-3 cells were first labeled with 1 μM green fluorescence dye carboxyfluorescein diacetate succinimidyl ester (CFSE,^#^65-0850, eBioscience, ThermoFisher scientific, Shanghai, China), followed by incubation with γδ T cells according to designated E (effector, γδ T): T (target, BxPC-3) ratios (1:1, 10:1, and 20:1). After 6 hours, incubated BxPC-3 cells and γδ T cells were collected and stained with propidium iodide (PI, ^#^V13242, eBioscience, ThermoFisher scientific, Shanghai, China) for 5 min at room temperature, followed by flow cytometry analysis. The apoptotic BxPC-3 cells were gated from both positive CFSE and PI signals.

**CA19-9 detection**

The serum concentration of CA19-9 was evaluated by a chemiluminescent immunoassay before treatment and 90 days after treatment.

**CTCs analysis**

Peripheral blood (7.5 mL) was obtained from patients before treatment and 90 days after treatment for the detection of CTCs. The samples were stored at room temperature and processed within 6 h of collection. Mononuclear cells were separated from other blood components using a human PBMC separation liquid (^#^LDS1075CB, Tianjin Haoyang Biological

Manufacture Co., Ltd., Tianjin, China). The cells were centrifuged at 1,800 ×g for 20 min at 4°C and washed twice with sterile Hank’s balanced salt solution (^#^24020117, Life Technologies, Carlsbad, CA, USA). The isolated cells were enriched by magnetic CD326 (epithelial cell adhesion molecule) MicroBeads (Miltenyi Biotech, Bergisch Gladbach, Germany). Then, a phycoerythrin (PE)-labeled antibody against CD45 (^#^5B1, 10 μL), fluorescein isothiocyanate (FITC)-labeled antibodies against cytokeratins 8, 18, and 19 (^#^REA885, 10 μL), and an allophycocyanin (APC)-labeled antibody against CD326 (^#^HEA-125, 10 μL) (Miltenyi Biotech) were added to the enriched cells, followed by incubation in the dark for 12 min at room temperature. The cell pellets were resuspended in 500 μL of PBS, and the samples were analyzed by a FACSCanto^TM^ II flow cytometer (Becton Dickinson, Franklin Lakes, NJ, USA) with a CD45^-^/CK^+^/CD326^+^ gating strategy. The absolute number of CD45^-^CK^+^CD326^+^ cells was used to quantitate the CTC levels.

**Immune parameters**

The immune parameters mainly included T cells, NK cells, and Th1 cytokines. Two milliliters of peripheral blood was obtained from patients before treatment and 90 days after treatment for the detection of immune function by flow cytometry with a FACSCantoTM II (BD Biosciences, San Jose, CA, USA). The multitest 6-color TBNK reagent (^#^337166, BD Biosciences) with Trucount tubes were used to detect the absolute numbers of CD3^+^CD4^+^ cells, CD3^+^CD8^+^ cells, total CD3^+^ cells, and CD3^-^CD16^+^CD56^+^ cells. For cytokines detection, a human Th1/Th2 cytokine kit II (^#^551809, BD Biosciences) for a cytometric bead array was used to detect the expression of IL-2, TNF-β and IFN-γ.

**Surface receptors detection**

Five milliliters of peripheral blood was obtained from patients before treatment and 90 days after treatment for the detection of surface receptors in γδ T cells by flow cytometry with a FACSCanto^TM^ II (BD Biosciences, San Jose, CA, USA). The staining and detection of surface receptors were performed according to product instructions. Briefly, cells were collected, washed once with PBS buffer and stained with corresponding antibodies for 20 minutes in the dark at room temperature before detection, followed by flow cytometry analysis. The monoclonal antibodies of mouse anti-human NKG2D (Biolegend, ^#^130212), PD-1 (Biolegend, ^#^135217), and CD44 (Biolegend, ^#^338804) were used.

**PS score**

PS score was used to assess the quality of life (QOL). PS scores were calculated by medical staffs’ observation before and 3 months after treatment.

**TMTD**

The tumor sizes of the enrolled patients were assessed by CT examination in accordance with RECIST v1.1 criteria.

**REFERENCES**

1. Lin, M. *et al.* Pembrolizumab plus allogeneic NK cells in advanced non-small cell lung cancer patients. *J Clin Invest*. **130**, 2560-2569 (2020).

2. Alnaggar, M. *et al.* Allogenic Vgamma9Vdelta2 T cell as new potential immunotherapy drug for solid tumor: a case study for cholangiocarcinoma. *J Immunother Cancer*. **7**, 36 (2019).

**Figure. S1.**

**
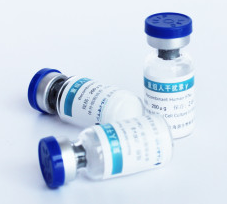

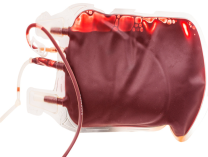

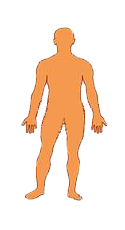

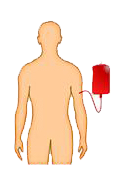

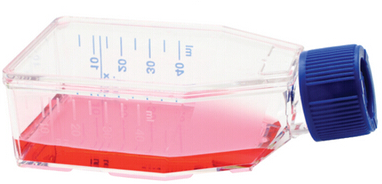
**

Healthy donors

Patients

Drawing blood from donors

PBMC isolation

γδ T cell culture

γδ T cell amplification

**γδ T cell**

Cell quality detection

γδ T cell infusion


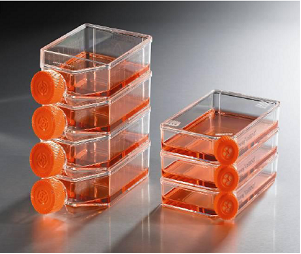


Adding growth factors

**Supplementary Fig. S1 Flow chart of preparation and infusion of γδ T cells from healthy donors into LAPC patients (*n* = 30).**

**Figure. S2.**

**
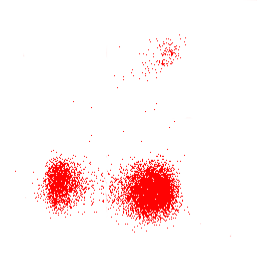

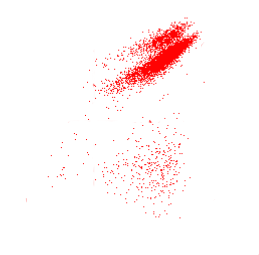
**

**Vδ2**

**Vδ2**

-10^-2^

0

10^3^

10^4^

10^5^

0

10^3^

10^4^

10^5^

-10^3^

Q1

Q2

Q4

Q3

0.08

1.92

26.9

71.1

-10^-2^

0

10^3^

10^4^

10^5^

0

10^3^

10^4^

10^5^

-10^3^

Q1

Q2

Q4

Q3

94.6

0.34

4.96

0.1

**CD3**

**CD3**

**a**

**b**

**Supplementary Fig. S2 The proportion of γδ T cells before expansion and on day 12 after expansion from a representative donor (*n* = 1)**.

**Figure. S3.**

**Cytotoxicity (%)**

0

20

40

60

80

100

***

##

1:1

10:1

20:1

**Ratio of γδ T to BxPC-3 cells**

**Supplementary Fig. S3 Cytotoxicity of the expanded γδ T cells.** The effector cells were γδ T cells derived from donor PBMCs and the target cells were BxPC-3 cells; the effector:target (E/T) ratios were 1:1, 10:1, and 20:1. All experiments were performed in triplicate. *n* = 10. Data are shown as individual data points with scatter dot plot (mean with SEM). Comparison between 10:1 and 1:1, ****P*<0.001; comparison between 10:1 and 20:1, ^##^*P*<0.01; Two-tailed Student’s *t* test.

**Figure. S4.**

**
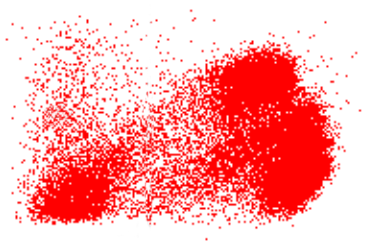

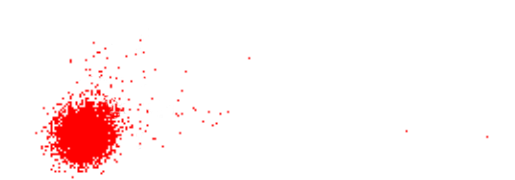

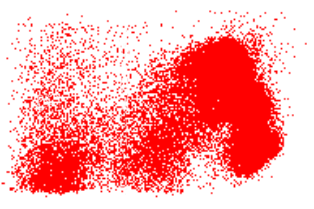

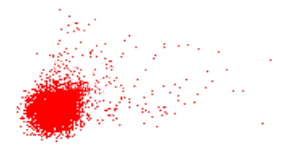
**

**CK FITC-A**

**SSC-A**

**CK FITC-A**

**SSC-A**

0

10^2^

10^3^

10^4^

10^5^

10^2^

0

10^3^

10^4^

10^5^

**CD326 APC-A**

0

10^1^

10^2^

10^3^

10^4^

10^5^

0

10^2^

10^3^

10^4^

10^5^

**CD45 PE-A**

P2

Q1

Q2

Q3

Q4

0

10^2^

10^3^

10^4^

10^5^

10^2^

0

10^3^

10^4^

10^5^

**CD326 APC-A**

0

10^1^

10^2^

10^3^

10^4^

10^5^

0

10^2^

10^3^

10^4^

10^5^

**CD45 PE-A**

Q1

Q2

Q3

Q4

P2

**c**

**a**

**b**

**d**

**Supplementary Fig. S4 CTC analysis in a representative patient who received combination therapy.** (a) The number of CD45^-^ cells (P2 gate) before combination therapy. (b) The number of CTCs (CD45^-^CK^+^CD326^+^, Q2 gate) before combination therapy. (c) The number of CD45^-^ cells (P2 gate) after combination therapy. (d) The number of CTCs (CD45^-^CK^+^CD326^+^, Q2 gate) after combination therapy (*n* = 1).

**Figure. S5.**

Cumulative survival

Cumulative survival

0

6

12

18

24

30

36

42

Group A

Group B

Median OS from diagnosis (months)

**a**

0

100

Median PFS from diagnosis (months)

**b**

Group A

Group B

*P* = 0.03

HR, 0.58 (95% CI, 0.34-0.99)

0

6

12

18

24

30

36

42

*P* = 0.03

HR, 0.59 (95% CI, 0.35-1.00)

20

40

60

80

0

100

20

40

60

80

**Supplementary Fig. S5 Kaplan-Meier survival curves.** (a) Graph showed OS from the time of diagnosis in total population (*n* = 62). (b) Graph showed PFS from the time of diagnosis in total population (*n* = 62).

**Figure. S6.**

**
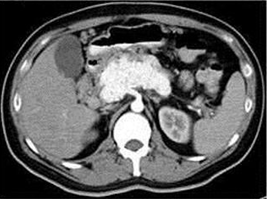

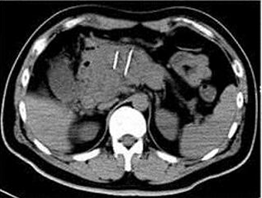

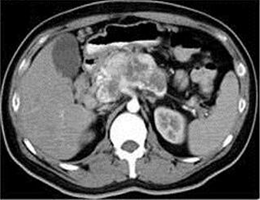
**

Pre-operation

During operation

Post-operation

**a**

**b**

**c**

**Supplementary Fig. S6 Representative CT images of a patient from group A.** (a) a contrast-enhanced CT scan taken showed a 5.4 × 4.1 cm contrast-enhanced lesion (red arrow) in the neck and body of the pancreas before IRE. (b) Two IRE electrodes were inserted into the tumor. (c) After IRE-γδ T treatment 3 months, CT scan showed a 5.3 × 4.0 cm lesion with a large area of necrosis in the neck and body of the pancreas (*n* = 1).

**Table S1. The clinical pathological data of the patients who received IRE plus allogeneic Vγ9Vδ2 T cells (group A) or IRE alone (group B)**

| **Patient no.** | | **Age** | **Sex** | **IHC result** | | | | | | | | | | **Pathological type** |
| --- | --- | --- | --- | --- | --- | --- | --- | --- | --- | --- | --- | --- | --- | --- |
|  |  |  |  | CK7 | CEA | Villin | CK19 | CK20 | Ki67 | Syn | CgA | EMA | P504 |  |
| Group A | 1 | 32 | Male | + | + | + | + | - | ND | ND | ND | ND | ND | Poorly differentiated adenocarcinoma |
|  | 2 | 43 | Female | + | + | + | + | + | ND | ND | ND | ND | ND | Moderately differentiated adenocarcinoma |
|  | 3 | 75 | Male | + | + | + | + | ND | ND | ND | ND | ND | ND | Poorly differentiated adenocarcinoma |
|  | 4 | 21 | Male | + | + | + | + | ND | >1%+ | ND | ND | ND | ND | Moderately-poorly differentiated adenocarcinoma |
|  | 5 | 74 | Female | + | + | + | + | - | ND | ND | ND | ND | ND | Poorly differentiated adenocarcinoma |
|  | 6 | 79 | Male | + | + | + | + | - | >10%+ | ND | ND | ND | ND | Moderately differentiated adenocarcinoma |
|  | 7 | 44 | Female | + | + | + | + | + | ND | ND | ND | ND | ND | Moderately differentiated adenocarcinoma |
|  | 8 | 52 | Male | + | + | - | + | - | ND | ND | ND | ND | ND | Poorly differentiated adenocarcinoma |
|  | 9 | 61 | Male | + | + | + | + | - | >50%+ | ND | ND | ND | ND | Moderately differentiated adenocarcinoma |
|  | 10 | 23 | Male | + | + | + | + | + | >10%+ | ND | ND | ND | ND | Poorly differentiated adenocarcinoma |
|  | 11 | 38 | Female | + | + | + | + | + | >50%+ | ND | ND | ND | ND | Poorly differentiated adenocarcinoma |
|  | 12 | 40 | Female | + | + | + | + | - | ND | ND | ND | ND | ND | Poorly differentiated adenocarcinoma |
|  | 13 | 48 | Male | + | + | + | + | - | ND | ND | ND | ND | ND | Poorly differentiated adenocarcinoma |
|  | 14 | 50 | Male | + | + | - | ND | - | ND | ND | - | + | ND | Poorly differentiated adenocarcinoma |
|  | 15 | 56 | Female | + | + | + | + | - | ND | - | ND | ND | ND | Poorly differentiated adenocarcinoma |
|  | 16 | 63 | Female | + | + | + | + | - | >10%+ | ND | ND | ND | ND | Moderately differentiated adenocarcinoma |
|  | 17 | 66 | Male | + | + | + | + | - | ND | ND | ND | ND | ND | Poorly differentiated adenocarcinoma |
|  | 18 | 70 | Male | + | + | + | + | + | >10%+ | ND | ND | ND | ND | Moderately-poorly differentiated adenocarcinoma |
|  | 19 | 73 | Male | + | + | + | + | ND | >50%+ | ND | ND | ND | ND | Poorly differentiated adenocarcinoma |
|  | 20 | 65 | Female | + | + | - | + | + | 10%+ | ND | ND | ND | ND | Moderately differentiated adenocarcinoma |
|  | 21 | 72 | Male | + | + | - | + | ND | >10%+ | ND | ND | ND | ND | Poorly differentiated adenocarcinoma |
|  | 22 | 78 | Female | + | + | + | + | - | ND | ND | ND | ND | ND | Moderately differentiated adenocarcinoma |
|  | 23 | 76 | Male | + | + | + | ND | - | 30%+ | ND | ND | ND | ND | Poorly differentiated adenocarcinoma |
|  | 24 | 58 | Female | + | + | - | + | - | ND | ND | ND | ND | ND | Poorly differentiated adenocarcinoma |
|  | 25 | 62 | Male | + | + | - | + | - | 5%+ | ND | ND | ND | ND | Poorly differentiated adenocarcinoma |
|  | 26 | 63 | Female | + | + | + | + | + | >30%+ | ND | ND | ND | ND | Poorly differentiated adenocarcinoma |
|  | 27 | 68 | Female | - | + | - | + | ND | ND | ND | - | ND | ND | Moderately differentiated adenocarcinoma |
|  | 28 | 70 | Male | + | + | + | + | ND | ND | - | ND | ND | ND | Poorly differentiated adenocarcinoma |
|  | 29 | 74 | Female | + | + | + | ND | ND | 50%+ | ND | ND | ND | ND | Moderately differentiated adenocarcinoma |
|  | 30 | 78 | Male | + | + | + | + | ND | ND | ND | - | ND | ND | Poorly differentiated adenocarcinoma |
| Group B | 31 | 23 | Male | + | + | + | + | + | >30%+ | ND | ND | ND | ND | Moderately differentiated adenocarcinoma |
|  | 32 | 43 | Female | + | + | + | + | ND | >10%+ | ND | ND | ND | ND | Poorly differentiated adenocarcinoma |
|  | 33 | 60 | Male | + | + | + | + | - | ND | - | ND | ND | ND | Poorly differentiated adenocarcinoma |
|  | 34 | 61 | Male | + | + | + | + | + | 60%+ | ND | ND | ND | ND | Poorly differentiated adenocarcinoma |
|  | 35 | 28 | Male | + | + | + | + | - | 50%+ | ND | ND | ND | ND | Poorly differentiated adenocarcinoma |
|  | 36 | 36 | Female | + | + | - | ND | - | 30%+ | ND | ND | ND | ND | Poorly differentiated adenocarcinoma |
|  | 37 | 38 | Male | + | + | + | + | + | 50%+ | ND | ND | ND | ND | Poorly differentiated adenocarcinoma |
|  | 38 | 40 | Female | + | + | - | ND | + | 50%+ | ND | ND | ND | ND | Moderately differentiated adenocarcinoma |
|  | 39 | 45 | Female | + | + | + | + | + | ND | ND | - | ND | ND | Moderately differentiated adenocarcinoma |
|  | 40 | 71 | Male | + | + | - | ND | - | 30%+ | ND | ND | ND | ND | Poorly differentiated adenocarcinoma |
|  | 41 | 74 | Female | + | + | + | ND | - | 5%+ | ND | ND | ND | ND | Poorly differentiated adenocarcinoma |
|  | 42 | 80 | Male | + | + | + | + | + | >20%+ | ND | ND | ND | ND | Poorly differentiated adenocarcinoma |
|  | 43 | 48 | Female | + | + | + | + | ND | ND | ND | ND | ND | - | Poorly differentiated adenocarcinoma |
|  | 44 | 50 | Male | + | - | - | + | - | ND | ND | ND | ND | ND | Poorly differentiated adenocarcinoma |
|  | 45 | 52 | Female | + | + | + | + | ND | >50%+ | ND | ND | ND | ND | Poorly differentiated adenocarcinoma |
|  | 46 | 74 | Male | + | + | + | ND | + | 30%+ | ND | ND | ND | ND | Moderately-poorly differentiated adenocarcinoma |
|  | 47 | 77 | Male | + | + | + | ND | - | >60%+ | ND | ND | ND | ND | Poorly differentiated adenocarcinoma |
|  | 48 | 80 | Female | + | + | + | + | - | >20%+ | ND | ND | ND | ND | Poorly differentiated adenocarcinoma |
|  | 49 | 56 | Male | + | + | + | + | ND | ND | - | ND | ND | ND | Moderately differentiated adenocarcinoma |
|  | 50 | 58 | Female | + | + | + | + | - | 20%+ | ND | ND | ND | ND | Poorly differentiated adenocarcinoma |
|  | 51 | 66 | Male | + | + | + | + | ND | >10%+ | ND | ND | ND | ND | Poorly differentiated adenocarcinoma |
|  | 52 | 73 | Female | + | + | + | + | - | >10%+ | ND | ND | ND | ND | Poorly differentiated adenocarcinoma |
|  | 53 | 76 | Male | + | + | - | + | - | 10%+ | ND | ND | ND | ND | Poorly differentiated adenocarcinoma |
|  | 54 | 79 | Male | + | + | + | ND | + | 60%+ | ND | ND | ND | ND | Poorly differentiated adenocarcinoma |
|  | 55 | 59 | Male | + | + | + | + |  | >80%+ | ND | ND | ND | ND | Poorly differentiated adenocarcinoma |
|  | 56 | 61 | Female | + | + | + | + | - | >20%+ | ND | ND | ND | ND | Moderately differentiated adenocarcinoma |
|  | 57 | 68 | Male | + | + | + | + | ND | >10%+ | ND | ND | ND | ND | Poorly differentiated adenocarcinoma |
|  | 58 | 60 | Female | + | + | + | + | - | >10%+ | ND | ND | ND | ND | Poorly differentiated adenocarcinoma |
|  | 59 | 72 | Male | + | + | + | + | + | >5%+ | ND | ND | ND | ND | Poorly differentiated adenocarcinoma |
|  | 60 | 70 | Female | + | + | + | ND | - | 30%+ | ND | ND | ND | ND | Moderately differentiated adenocarcinoma |
|  | 61 | 79 | Male | + | + | + | ND | + | 70%+ | ND | ND | ND | ND | Moderately differentiated adenocarcinoma |
|  | 62 | 75 | Male | + | + | - | + | - | 10%+ | ND | ND | ND | ND | Moderately-poorly differentiated adenocarcinoma |

Note: Range of specimen diameter = 0.1~0.2 cm; IHC = immunohistochemistry.

**Table S2. The quality control criteria of γδ T cells on day 9 of culture**

| **Indicators** | **Criteria** |
| --- | --- |
| Live cells | ≥90% |
| CD3^+^Vδ2^+^ cells | ≥70% |
| Endotoxin | ≤1.0 EU/ml |
| Cytotoxicity | ≥70% |
| Sterile test | Negative for bacterial, fungal and mycoplasma contamination |

**Table S3. The release criteria of γδ T cells on day 12 of culture**

| **Indicators** | **Criteria** |
| --- | --- |
| Live cells | ≥90% |
| CD3^+^Vδ2^+^ cells | ≥85% |
| Endotoxin | ≤1.0 EU/ml |
| Sterile test | Negative for bacterial, fungal and mycoplasma contamination |

**Table S4. Adverse events observed in 34 of the 62 participants**

| **Adverse event** | **Grade I/II** | **Grade III** | **Grade IV** | **Treatment** |
| --- | --- | --- | --- | --- |
| **Gastrointestinal** |  |  |  |  |
| Loss of appetite | 3 | 1 | 0 | Nasojejunal tube feeding |
| Nausea | 2 | 0 | 0 | Antiemetics |
| Vomiting | 3 | 0 | 0 | Antiemetics, nasojejunal tube feeding |
| Gastroparesis | 2 | 3 | 0 | Conservative |
| Diarrhea | 3 | 0 | 0 | Pancreatic enzyme suppletion, loperamide |
| **Infection** |  |  |  |  |
| Pancreatic fistula | 0 | 1 | 0 | Drainage and antibiotics |
| Pancreatitis | 0 | 2 | 1 | Drainage and antibiotics |
| Abscess | 3 | 1 | 0 | Antibiotics |
| **Biliary** |  |  |  |  |
| Obstruction | 0 | 1 | 0 | ERCP and stent placement |
| Cholangitis | 0 | 2 | 1 | PTCD and antibiotics |
| **Vascular** |  |  |  |  |
| Portal vein thrombosis | 2 | 0 | 0 | Anticoagulants |
| Bleeding from duodenal ulcer | 0 | 0 | 1 | Endoscopy and blood transfusion |
| **Other** |  |  |  |  |
| Pain | 4 | 0 | 0 | Analgesics |
| Cardiac arrhythmias | 3 | 0 | 0 | Resolved spontaneously |
| **Total** | 25 | 11 | 3 |  |

Note: ERCP = endoscopic retrograde cholangiopancreatography, PTCD = percutaneous transhepatic cholangiography drain
